# Evaluation of the efficacy and safety of intravenous remdesivir in adult patients with severe COVID-19: study protocol for a phase 3 randomized, double-blind, placebo-controlled, multicentre trial

**DOI:** 10.1186/s13063-020-04352-9

**Published:** 2020-05-24

**Authors:** Yeming Wang, Fei Zhou, Dingyu Zhang, Jianping Zhao, Ronghui Du, Yi Hu, Zhenshun Cheng, Ling Gao, Yang Jin, Guangwei Luo, Shouzhi Fu, Qiaofa Lu, Guanhua Du, Ke Wang, Yang Lu, Guohui Fan, Yi Zhang, Ying Liu, Shunan Ruan, Wen Liu, Thomas Jaki, Frederick G. Hayden, Peter W. Horby, Bin Cao, Chen Wang

**Affiliations:** 1 Department of Pulmonary and Critical Care Medicine, Center of Respiratory Medicine, National Clinical Research Center for Respiratory Diseases, China-Japan Friendship Hospital, Beijing, China; 2 Jin Yin-tan Hospital, Wuhan, Hubei Province China; 3 Tongji Hospital, Tongji Medical College of Huazhong University of Science & Technology, Wuhan, China; 4 Wuhan Lung Hospital, Wuhan, China; 5 The Central Hospital of Wuhan, Wuhan, China; 6 Zhongnan Hospital of Wuhan University, Wuhan, China; 7 Renmin Hospital of Wuhan University, Wuhan, China; 8 Union Hospital, Tongji Medical College of Huazhong University of Science & Technology, Wuhan, China; 9 Wuhan First Hospital, Wuhan, China; 10 Wuhan Third Hospital, Wuhan, China; 11 Wuhan Fourth Hospital, Wuhan, China; 12 Institute of Medicine, Chinese Academy of Medical Sciences, Beijing, China; 13 Institute of Clinical Medical Sciences, China-Japan Friendship Hospital, Beijing, China; 14 Lancaster University, Lancaster, UK; 15 University of Virginia School of Medicine, Charlottesville, VA USA; 16 ISARIC, University of Oxford, Oxford, UK; 17 Institute of Respiratory Medicine, Chinese Academy of Medical Sciences, Beijing, China; 18 Peking Union Medical College, Beijing, China

**Keywords:** COVID-19, Clinical trial, Remdesivir, Antiviral, China, Administrative information

## Abstract

**Background:**

Coronavirus disease 2019 (COVID-19), caused by a novel corinavirus (later named SARS-CoV-2 virus), was fistly reported in Wuhan, Hubei Province, China towards the end of 2019. Large-scale spread within China and internationally led the World Health Organization to declare a Public Health Emergency of International Concern on 30^th^ January 2020. The clinical manifestations of COVID-19 virus infection include asymptomatic infection, mild upper respiratory symptoms, severe viral pneumonia with respiratory failure, and even death. There are no antivirals of proven clinical efficacy in coronavirus infections. Remdesivir (GS-5734), a nucleoside analogue, has inhibitory effects on animal and human highly pathogenic coronaviruses, including MERS-CoV and SARS-CoV, in in vitro and in vivo experiments. It is also inhibitory against the COVID-19 virus in vitro. The aim of this study is to assess the efficacy and safety of remdesivir in adult patients with severe COVID-19.

**Methods:**

The protocol is prepared in accordance with the SPIRIT (Standard Protocol Items: Recommendations for Interventional Trials) guidelines. This is a phase 3, randomized, double-blind, placebo-controlled, multicentre trial. Adults (≥ 18 years) with laboratory-confirmed COVID-19 virus infection, severe pneumonia signs or symptoms, and radiologically confirmed severe pneumonia are randomly assigned in a 2:1 ratio to intravenously administered remdesivir or placebo for 10 days. The primary endpoint is time to clinical improvement (censored at day 28), defined as the time (in days) from randomization of study treatment (remdesivir or placebo) until a decline of two categories on a six-category ordinal scale of clinical status (1 = discharged; 6 = death) or live discharge from hospital. One interim analysis for efficacy and futility will be conducted once half of the total number of events required has been observed.

**Discussion:**

This is the first randomized, placebo-controlled trial in COVID-19. Enrolment began in sites in Wuhan, Hubei Province, China on 6^th^ February 2020.

**Trial registration:**

ClinicalTrials.gov: NCT04257656. Registered on 6 February 2020.

## Introduction

### Background and rationale {6a}

In December 2019, Wuhan City, Hubei Province experienced an outbreak of pneumonia of unknown cause. On 7^th^ January 2020, a previously unidentified betacoronavirus, later named SARS-CoV-2 virus (or the disease named COVID-19), was identified by the Chinese Center for Disease Control and Prevention (China CDC) as the aetiological agent [1]. The SARS-CoV-2 probably derived originally from bats, and amongst coronaviruses known to infect humans, is most closely related to, but distinct from, the SARS coronavirus. The clinical manifestations of COVID-19 virus infection include asymptomatic infection, mild upper respiratory symptoms, severe viral pneumonia with respiratory failure, and even death. Although the risk of severe illness is not yet clear, hospitals in areas with significant community transmission have experienced a major increase in the number of hospitalized pneumonia patients, with the frequency of severe disease in hospitalized patients being as high as 30% [2–4]. The progression from prodromes (usually fever, fatigue, and cough) to severe pneumonia requiring oxygen support, mechanical ventilation, or extracorporeal membrane oxygenation (ECMO) is most commonly seen in the second week following onset of symptoms of a viral infection [2]. The kinetics of viral replication in the respiratory tract has not been well characterized, but this relatively slow progression provides a potential time window and opportunity for antiviral therapies to influence the course of the disease.

Remdesivir (GS-5734) is a monophosphoramidate prodrug of an adenosine analogue (GS-441524) and has broad action against a range of RNA viruses [5]. It has been identified as the most promising therapeutic agent for evaluation in the treatment of COVID-19 by an expert committee convened by the WHO R&D Blueprint [6]. The primary mechanism of action is the intracellular incorporation of the pharmacologically active nucleoside triphosphate form into nascent RNA chains by the viral RNA-dependent RNA polymerase, causing premature RNA chain termination [7–9]. In vitro experiments have shown that remdesivir inhibits bat coronaviruses, endemic human coronavirus (OC43, 229E), and the human pathogenic coronaviruses MERS-CoV, SARS-CoV, and COVID-19 [10–13]. Remdesivir has shown preventive and therapeutic effects in a mouse model of SARS-CoV [10]. In a MERS-CoV mouse model, prophylactic and therapeutic administration of remdesivir improved lung function, decreased lung viral load, and reduced severe lung pathological findings [14]. Remdesivir has also shown prophylactic efficacy in MERS-CoV-infected Indian rhesus monkeys (personal communication: Gilead Sciences, Inc.).

Evaluation of intravenously administered remdesivir tolerance and safety in 94 healthy adult volunteers has found it to be generally well tolerated and to have an acceptable safety profile. The only significant adverse effects were transient grade 1 or grade 2 increases in aspartate transaminase (AST) and alanine transaminase (ALT) (personal communication: Gilead Sciences, Inc.). Further clinical experience was obtained through a randomized controlled trial in patients with Ebola virus disease. In this trial 175 patients received intravenous remdesivir with a loading dose on day 1 (200 mg in adults, and adjusted for body weight in pediatric patients), followed by a daily maintenance dose (100 mg in adults) starting on day 2 and continuing for 9 to 13 days. The only reported serious adverse event (SAE) was that of one patient who experienced hypotension that resulted in cessation of the remdesivir loading dose, followed rapidly by cardiac arrest. However, the cause of death could not be distinguished from the patient’s underlying severe Ebola virus disease [15].

Given the in vitro and in vivo effectiveness of remdesivir against a range of pathogenic human coronaviruses and the acceptable tolerance and safety in humans, we developed this trial with the objective of evaluating the safety and efficacy of intravenous remdesivir in adults with severe COVID-19.

## Objectives {7}

The trial objective is to evaluate the efficacy and safety of intravenous remdesivir combined with standard of care compared with standard care alone in adult patients with severe COVID-19.

### Trial design {8}

This is a phase 3, parallel group, randomized, double-blind, placebo-controlled, superiority, multicentre trial. The allocation ratio is 2:1 in favour of remdesivir to maximize learning about the experimental treatment whilst allowing a wider pool of patients access to the experimental treatment in order to support recruitment.

## Methods: participants, interventions, and outcomes

### Study setting {9}

The setting involved hospitals in Wuhan city, Hubei Province, People’s Republic of China. Study sites included Jin Yin-tan Hospital, Tongji Hospital, Tongji Medical College of Huazhong University of Science & Technology, Wuhan Lung Hospital, the Central Hospital of Wuhan, Zhongnan Hospital of Wuhan University, Renmin Hospital of Wuhan University, Union Hospital, Tongji Medical College of Huazhong University of Science & Technology, Wuhan First Hospital, Wuhan Third Hospital, and Wuhan Fourth Hospital.

### Eligibility criteria {10}

#### Inclusion criteriaThe inclusion criteria are as follows:

1. Age ≥ 18 years at time of signing of informed consent form
2. Laboratory (reverse transcription polymerase chain reaction [RT-PCR])-confirmed COVID-19
3. Pneumonia confirmed with chest imaging
4. Hospitalized with SPO2 ≤ 94% on room air or PaO2/FiO2 ≤ 300 mmHg
5. ≤ 12 days since symptoms onset
6. Willingness of study participant to accept randomization to any assigned treatment arm
7. Males and females of child-bearing age must agree to use effective birth control measures (hormone method, barrier method, or abstinence) during the trial and at least 7 days after the last medication dose
8. Must agree not to enrol in any other study of an antiviral agent prior to completing the 28-day follow-up.

#### Exclusion criteriaThe exclusion criteria are as follows:

1. Physician makes a decision that trial involvement is not in patient’s best interest, or patient has any condition that does not allow the protocol to be followed safely
2. Known severe liver disease (e.g. cirrhosis, ALT > 5 × upper limit of normal (ULN), or AST > 5 × ULN)
3. Patients who are pregnant or breastfeeding, or positive pregnancy test in women of childbearing age
4. Patients with known severe renal impairment (estimated glomerular filtration rate ≤ 30 ml/min/1.73 m^2^), or patients receiving continuous renal replacement therapy, haemodialysis, or peritoneal dialysis
5. Will be transferred to any other hospital which is not a study site within 72 h
6. Receipt of any investigational treatment for COVID-19 within 30 days prior to screening.

#### Who will take informed consent? {26a}

Informed consent will be obtained from eligible patients or their substitute decision-makers (for patients lacking decision-making capacity) by study physicians or other trial staff with delegated responsibility.

### Additional consent provisions for collection and use of participant data and biological specimens {26b}

The consent form includes provisions for research data and samples and residual clinical blood samples to be stored for future scientific research on COVID-19.

### Interventions

#### Explanation for the choice of comparators {6b}

The active arm is intravenously administered remdesivir in addition to routine supportive care. Remdesivir was chosen for evaluation because of its in vitro activity against SARS-CoV, MERS-CoV, and SARS-CoV-2, and its in vitro activity against SARS-CoV and MERS-CoV. There are safety data for remdesivir in healthy human volunteers and individuals with Ebola virus disease. These data suggest that remdesivir is generally well tolerated and has an acceptable safety profile. It has been identified as the most promising therapeutic agent for treatment of COVID-19 by the WHO R&D Blueprint [6]. The dose of remdesivir was selected on the recommendation of Gilead Sciences, Inc., based on known pharmacokinetics and dose-ranging studies in healthy volunteers. The control arm is placebo in addition to routine supportive care. There are no proven antiviral therapies for SARS-CoV-2 to include as an active control comparator.

#### Intervention description {11a}

#### The intervention is described as follows for the experimental and control groups:

1. Group A (experimental group)
  - Loading dose: Remdesivir 200 mg in 350 ml normal saline (0.9% sodium chloride) single daily dose infused intravenously over approximately 30–60 min (with a target time of 30 min) for 1 day
  - Maintenance dose: Remdesivir 100 mg in 250 ml normal saline (0.9% sodium chloride) single daily dose infused intravenously over approximately 30–60 min (with a target time of 30 min) for 9 days.
2. Group B (control group)
  - Loading dose: Placebo in 350 ml normal saline (0.9% sodium chloride) single daily dose infused intravenously over approximately 30–60 min (with a target time of 30 min) for 1 day
  - Maintenance dose: Placebo in 250 ml normal saline (0.9% sodium chloride) single daily dose infused intravenously over approximately 30–60 min (with a target time of 30 min) for 9 days.

### Criteria for discontinuing or modifying allocated interventions {11b}

The discontinuation/modification criteria are as follows:

1. Any serious or intolerable adverse event (AE) which, in the investigators’ judgement, requires withdrawal of the subject from the study. This may include drug intolerance or an unacceptable AE; any severe grade 4 AE, regardless of whether the AE is considered likely related to the study drug or not; significant results of laboratory tests which may require withdrawal of the participant, at the discretion of the researchers and the clinician; allergic reactions (including oropharyngeal oedema, severe rash, bronchospasm, and immediate-type allergic reactions); acute hepatitis;^1^ stage III or above acute kidney injury
2. Investigator request for the subject to be withdrawn from the study.

### Strategies to improve adherence to interventions {11c}

This item is not applicable, since active agent and placebo are administrated intravenously by healthcare professionals.

### Relevant concomitant care permitted or prohibited during the trial {11d}

This study seeks to evaluate the effect of remdesivir in addition to standard of care alone, rather than an effect that might be altered by other experimental treatments. Therefore, all concomitant care and interventions are permitted other than concomitant receipt of any other experimental treatment.

#### Provisions for post-trial care {30}

No special arrangements for post-trial care are anticipated.

### Outcomes {12}

#### Primary outcome measure

The primary outcome measure is time to clinical improvement (TTCI) (censored at day 28).

TTCI is defined as the time (in days) from randomization of study treatment (active or placebo) until a decline of two categories on a six-category ordinal scale of clinical status (1 = hospital discharge; 2 = hospitalization, not requiring supplemental oxygen; 3 = hospitalization, requiring supplemental oxygen (but not non-invasive mechanical ventilation [NIV]/high-flow nasal cannula [HFNC] therapy); 4 = hospitalization, requiring NIV and/or HFNC therapy; 5 = hospitalization, requiring ECMO and/or invasive mechanical ventilation [IMV]; 6 = death) or live hospital discharge, whichever comes first.

#### Secondary outcome measures:The secondary outcome measures are the following:

1. Percentage of subjects in each clinical category on the six-category ordinal scale (time frame: days 7, 14, 21, and 28)
2. Time to disease improvement, defined as the time to hospital discharge, or NEWS2 (National Early Warning Score 2) of ≤ 2 maintained for 24 h (time frame: up to 28 days)
3. All-cause mortality (time frame: up to 28 days)
4. Duration (days) of IMV (time frame: up to 28 days)
5. Duration (days) of supplemental oxygenation (time frame: up to 28 days)
6. Length of hospital stay (days) (time frame: up to 28 days)
7. Incidence of nosocomial secondary infection
8. Time to SARS-CoV-2 virus RT-PCR negativity in upper and lower respiratory tract specimens (time frame: up to 28 days)
9. Change (reduction) in SARS-CoV02 viral RNA load in upper and lower respiratory tract specimens as assessed by area under viral load curve (time frame: up to 28 days)
10. Pharmacokinetic parameters of remdesivir or its active metabolites
11. Frequency of SAEs (time frame: up to 28 days).

### Participant timeline {13}

Table 1 shows the timeline of participant assessments/interventions.

**Table 1:** Study timeline of participant assessments/interventions

Process	Screening/baseline/randomization	Treatment phase	Follow-up phase		
	B/L/D0	D1–D10	D14 ±1	D21 ±3	D28 ±3
Inclusion/exclusion criteria	X				
Written informed consent	X				
Demographics	X				
Assessment of concomitant chronic diseases	X				
Efficacy/safety assessment					
ECG	X		X		
Chest imaging	X	X (only on D10)			X
Vital signs (body temperature, heart rate, blood pressure, breathing rate, oxygen saturation, etc.)	X	X	X	X	X
Clinical symptoms assessment (fever, cough, diarrhea, dyspnea)	X	X	X	X	X
Primary endpoint assessment	X	X	X	X	X
Adverse events	X	X	X	X	X
**Laboratory test**					
Diagnosis (clinical diagnosis, antibody diagnosis, or pathogen diagnosis)	X				
Whole blood count (sampling time)	X	X (only on D3, D7, and D10)			
Coagulation routine (sampling time)	X	X (only on D3, D7, and D10)			
Kidney and liver function tests (sampling time)	X	X (only on D3, D7, and D10)			
Arterial blood gas analysis	X				
Pregnancy test (urine/blood, for women of childbearing age only)	X				
Nasopharyngeal/ oropharyngeal swab	X	X	X	X	X
Lower respiratory tract specimen (sputum/tracheal aspirate/bronchial alveolar lavage fluid) if available	X	X	X	X	X
Feces/anal swabs	X	X	X	X	X
Pharmacokinetic sampling (approximately 4 ml whole blood)		X (only on D1, D3, D7 [as needed], and D10)			
**Interventions**					
Remdesivir/placebo		X			
Concomitant medications (record only)	X	X	X	X	X

### Sample size {14}

The primary outcome is TTCI (censored at day 28). An interim analysis for futility and efficacy is planned at the midpoint of the trial. At this point the sample size may also be re-assessed. A one-sided type I error of 2.5% across the two stages of the trial and a power of 80% if the hazard ratio comparing remdesivir to placebo is 1.4 is used. This hazard ratio corresponds to a reduction in TTCI to 15 days on remdesivir if the TTCI on standard of care was 21 days. Triangular boundaries [16] are used to account for the multiple looks at the data, and allowance for the 2:1 randomization in favour of remdesivir is made. Under these assumptions, the total number of events required in this study is 325.

Assuming an 80% event rate within 28 days across both arms and a dropout rate of 10% implies that approximately 453 patients are to be recruited for this trial (151 on placebo and 302 on remdesivir).

### Recruitment {15}

Patients will be recruited from the designated hospitals for patients with COVID-19 virus.

### Assignment of interventions: allocation

#### Sequence generation {16a}

A permuted block (30 patients per block) randomization sequence, including stratification, is prepared by a statistician not involved in the trial using SAS software, version 9.4 (SAS Institute). Patient randomization is stratified based on respiratory support methods at the time of enrolment: (1) no oxygen support, oxygen support with nasal duct or mask; (2) high-flow oxygen, NIV, IMV/ECMO.

#### Concealment mechanism {16b}

Eligible patients were allocated to receive medication in individually numbered packs according to the sequential order provided by the randomization centre (Jin Yin-tan Hospital central pharmacy). Remdesivir and placebo are pre-blinded and stored in a secure area in the pharmacy at a temperature strictly controlled according to the protocol. An independent pharmacist is assigned to dispense the study drug in waterproof, sealed, opaque bags.

#### Implementation {16c}

Patients will receive medication in individually numbered packs according to the sequential order provided by the randomization centre. Opaque envelopes were provided for emergency unblinding events. The allocation sequence was generated by the institutes of Materia Medica, Chinese Academy of Medical Sciences & Peking Union Medical College. Participants are enrolled by the investigators of each study site. A pharmacist in the central pharmacy assigns participants to interventions.

### Assignment of interventions: blinding

#### Who will be blinded? {17a}

This is a double-blind trial. Trial participants, investigators, care providers, outcome assessors, and data analysts are all blinded. Treatment allocation will only be unblinded after database lock.

### Procedure for unblinding if needed {17b}

Unblinding is permissible when investigators believe that there is a very strong need to know the study drug allocation in order to perform any agent treatment/action. Whenever possible, unblinding will only be conducted after discussion with the study principal investigator. The procedure for revealing a participant’s allocated intervention is as follows: (1) investigators confirm that the patient meets the criteria of unblinding according to the protocol; (2) the independent pharmacist opens the sealed envelope and informs the investigators of the allocation; (3) information about the date, time, and reason of unblinding will be recorded in the electronic data capture (EDC) system and the envelope; (4) the envelope must be sealed again as soon as possible and securely stored along with the primary files of the subject.

### Data collection and management

#### Plans for assessment and collection of outcomes {18a}

Investigators are responsible for assessment and collection of outcome, baseline, and other trial data, and the data are double checked by the clinical research coordinators and clinical research associates as well as data managers. Nasopharyngeal/oropharyngeal swabs, lower respiratory tract specimens (sputum/tracheal aspirate/bronchial alveolar lavage fluid), feces/anal swabs, and pharmacokinetic samples (whole blood) will be sent to a central laboratory, where tests will performed according to laboratory standard operating procedures (SOPs). Data collection forms can be found at https://edc.clinflash.net/login.

For the efficacy outcome measures, two scales (six-category ordinal scale of clinical status and NEWS2) are used. Investigators will be trained to use these scales.

#### Plans to promote participant retention and complete follow-up {18b}

Subjects may voluntarily withdraw from the trial at any time. This decision must be communicated with and reviewed by investigators. Staff at study sites should explain to these subjects the importance of staying in the study for the full duration of follow-up of this trial. For any withdrawal from this trial, investigators must fill in the withdrawal reason in the electronic case report form (eCRF) and try to complete all remaining assessments up to day 28 after randomization. For subjects who withdraw due to AEs, investigators should closely follow up their AEs until the AEs disappear, return to the baseline state, or the AE condition is stable. If subjects are lost to follow-up, existing data collected up until the time of loss to follow-up will be used.

Investigators may request for subjects to be withdrawn from receiving the study drugs for clinical or other reasons. In this case, subjects should remain in the trial after terminating the trial treatment and be followed up for all scheduled visits and the corresponding data recorded.

#### Data management {19}

The EDC system will be used for data management, including data entry, query, coding, security, and storage. The data manager built the database according to the protocol, and at the same time made logical verification settings for data validity, so as to verify the data. The data of each enrolled subject (including discontinued subjects) will be recorded into the database/eCRF rapidly, completely, and accurately. Each completed eCRF will be reviewed and signed (electronically) by the investigators. The data manager will check the data and ask investigators to resolve any queries identified. The data administrator will proofread and modify the data according to investigators’ answers. The data are securely stored in the EDC system, with the server located in Zhejiang Province, China.

### Confidentiality {27}

Subjects’ data collected in CRFs will be identified by a study subject ID only. Samples collected through the study will also be identified by a study subject ID only. The log linking the study subject ID with the patient identifying information will be held at each recruitment site. In the emergent or rare event that, for safety or regulatory reasons, it may be necessary to identify a subject, the sponsor and investigators were bound to keep this information confidential.

### Plans for collection, laboratory evaluation, and storage of biological specimens for genetic or molecular analysis in this trial/future use {33}

Nasopharyngeal/oropharyngeal swabs, lower respiratory tract specimens (sputum/tracheal aspirate/bronchial alveolar lavage fluid), feces/anal swabs, and pharmacokinetic samples (whole blood) will collected by investigators or designees, marked with the study subject ID, and then immediately sent to the central laboratory for coronavirus nucleic acid detection or remdesivir/active metabolites concentration detection. Sample collection, handling, labelling, storage, shipping, processing, etc. will be performed according to the requirements of the operation manual in the central laboratory, Teddy Clinical Research Laboratory (Shanghai) Ltd.

### Statistical methods

#### Statistical methods for primary and secondary outcomes {20a}

The primary outcome is TTCI up to day 28 of patients on remdesivir compared to patients receiving placebo. Patients will be randomized at a ratio of 2:1 to receive either remdesivir or placebo. All reporting will adhere to the Consolidated Standards of Reporting Trials (CONSORT) guideline as well as the corresponding extension for adaptive clinical trials [17, 18].

The final analysis of the primary endpoint will use a Cox proportional hazards model following the intention-to-treat principle. The final analysis will be conducted once a total of 325 events have occurred across both treatment groups. Under the assumption of an 80% event rate within 28 days and accounting for 10% dropout, this means that the maximum number of patients to be recruited is approximately 453. A standardized test statistic is then found on the basis of this model—if this test statistic exceeds 2.095, then it will be concluded that remdesivir is significantly better than placebo. Median unbiased effect estimates and confidence intervals accounting for the interim analysis will be reported [19]. Analysis of secondary endpoints will use a logistic model for binary variables such as mortality and a Cox proportional hazards model for time-to-event endpoints. A statistical analysis plan (SAP) providing all details of the analyses to be undertaken will be completed before any data analysis will be conducted.

### 3.18.2. Interim analyses {21b}

An interim analysis for efficacy and lack of benefit will be undertaken once a total of 163 events have been observed across all treatment group. For the interim analysis the primary endpoint will be analysed using a Cox proportional hazards model using the intention-to-treat principle. A standardized test statistic is then found on the basis of this model and if this test statistic falls below 0.741, the trial will be stopped for lack of benefit of remdesivir. If the test statistic exceeds 2.222, the trial will terminate with the conclusion, that remdesivir is significantly better than control. If the test statistics falls between 0.741 and 2.222, the trial continues to the second stage and a sample size re-estimation on the basis of the observed event rate will take place. The interim analysis is to be conducted by an independent statistician and reviewed by the DSMC. Should the trial stop for futility or efficacy the median unbiased estimate as well as confidence intervals accounting for the interim analysis will be reported [19].

### 3.18.3. Methods for additional analyses (e.g. subgroup analyses) {20b}

Separate supporting analysis will be undertaken for gender and age groups (<60 and over 60 years of age at randomization). These analyses will use a Cox proportional hazards model of the primary endpoint. Additionally, mortality will be analysed for each of these subgroups using a logistic regression model.

### 3.18.4. Methods in analysis to handle protocol non-adherence and any statistical methods to handle missing data {20c}

Multiple imputation will be used for missing data. The primary analysis will use the intention-to-treat principle and a per protocol analysis will be undertaken to assess the robustness of the findings.

### 3.18.5. Plans to give access to the full protocol, participant-level data, and statistical code {31c}

These plans are not yet in place.

### Oversight and monitoring

#### Composition of the coordinating centre and trial steering committee {5d}

##### 3.19.1.1. Coordinating centre

The study is led by the China-Japan Friendship Hospital, Wuhan Jin Yin-tan Hospital, Wuhan Central Hospital, Huazhong University of Science & Technology Tongji Hospital, Wuhan University Zhongnan Hospital, Wuhan Pulmonary Hospital, Wuhan University People's Hospital, Wuhan Union Hospital, and other hospitals.

#### 3.19.1.2. Trial steering committee

The trial steering committee consists of the following members:

- Chen Wang, President of Peking Union Medical College
- Bin Cao (China-Japan Friendship Hospital), deputy team leader of Novel Coronavirus Pneumonia (NCP) Group
- Dingyu Zhang (Jin Yin-tan Hospital), Director of NCP Designated Hospital.
- Ke Wang, Director of Institute of Medicine, Chinese Academy of Medical Sciences.

#### Trial operation committee

The trial operation committee consists of Yeming Wang and Fei Zhou of China-Japan Friendship Hospital, and Ying Liu, Shunan Ruan, and Wen Liu of Jin Yin-tan Hospital.

#### Trial monitoring

Trial monitoring is performed by Hangzhou Tigermed Consulting Co., Ltd.

#### Data management team

The data management team is Hangzhou Tigermed Consulting Co., Ltd.

#### Clinical research organization

The clinical research organization is Hangzhou Tigermed Consulting Co., Ltd.

### Composition of the data monitoring committee, its role and reporting structure {21a}

The independent Data Safety Monitoring Board (DSMB) in this study is responsible for reviewing the reports regarding the safety of the study patients and protocol adherence and making recommendations to continue or terminate the study or modify sample size on the basis of the results from the interim analysis. The DSMB members are all independent of the sponsor and have no financial or other conflicts of interest.

### Data monitoring committee (DMC) members

Table 2 lists the DMC members.

**Table 2:** Data monitoring committee members

Name	Role	Position
Jieming Qu	Chair	Professor of Respiratory Medicine & President, Ruijin Hospital Shanghai Jiao Tong University School of Medicine
Weichung Joe Shih	Member	Director of Biometrics Division of the Cancer Institute of New Jersey, Rutgers University
Rob Fowler	Member	Senior scientist, Evaluative Clinical Sciences, Trauma, Emergency & Critical Care Research Program, Sunnybrook Research Institute, Toronto, Canada
Rory Collins	Member	Head, Nuffield Department of Population Health, University of Oxford, UK
Chen Yao	Member	Biometrics Division of Peking University

### Adverse event reporting and harms {22}

AEs and SAEs will be collected from the time of informed consent to day 28. SAEs occurring after day 28 ± 3 will be reported if investigators determine that these SAEs are related to the study drugs. SAEs will be followed up until the SAE has subsided, returned to baseline, or is stable.

Investigators or designees are responsible for collecting, assessing, reporting, and managing AEs. AEs will be fully investigated and recorded in detail in the CRF, including onset date, the duration of AE, symptoms/signs, severity, action taken to manage the AE, relationship with the study drug, outcome of the AE, and date of outcome assessment (if outcome was other than recovering, not recovered, or unknown).

### Frequency and plans for auditing trial conduct {23}

The contract research organization (Hangzhou Tigermed Consulting Co., Ltd.) will be responsible for monitoring the trial. On-site monitoring and remote monitoring visits will be conducted in accordance with the study monitoring plan to ensure the completeness and accuracy of research data. Audits may be conducted at any time during or after the study.

### Plans for communicating important protocol amendments to relevant parties (e.g. trial participants, ethical committees) {25}

During the trial, the principal investigator will inform the Independent Ethics Committee (IEC) of any revision or modification of the protocol. The revision or modification will only be implemented after receiving IEC approval, unless it is necessary to be implemented in order to eliminate immediate and direct harm to patients, in which case the IEC will be informed as soon as possible.

After any protocol amendment, the informed consent form and any other written information provided to subjects will be updated as necessary. Investigators will inform subjects in time and ask them to sign the revised informed consent form to confirm their participation. The updated informed consent form must be approved by the IEC before implementation.

### Dissemination plans {31a}

We will communicate trial results to national and international health authorities, healthcare professionals, the public, and other relevant groups as soon as the trial results are available.

## Discussion

This study is the first double-blind, placebo-controlled trial of an experimental therapeutic for COVID-19. Whilst the COVID-19 epidemic has been declared a public health emergency of international concern, this does not negate the need to generate robust evidence in which healthcare professionals, patients, their families, and public health authorities can have confidence. In past epidemics, unproven treatments have been used on a compassionate use basis, in observational studies, or in underpowered trials, and the results are uncertainty and ongoing treatment dilemmas. Randomized, placebo-controlled trials are well established as the best way to minimize bias and confounding and produce reliable evidence. Such trials can be implemented even in the most difficult circumstances; in fact, the difficult circumstances themselves make it even more important that patient care is not driven by fear and rumour but by science and evidence. During health emergencies, doctors and politicians are under enormous pressure to find cures to save lives and stop the epidemic, whilst patients are willing to try anything in the face of a frightening and unknown threat. We must resist these pressures and make sure that patients benefit from the fruits of science, even in difficult times.

The use of placebo is especially beneficial in the midst of great anxiety, when the pressures to announce a cure or act on preliminary, but inconclusive data, are intense. A placebo relieves the temptation from all involved in the trial to engage in speculation and the worry that they are withholding what early (but often false) impressions may suggest is a promising drug. Whilst remdesivir has shown promising activity in preclinical models of severe CoV infection, it is currently unknown whether it will prove safe and effective in treating severe COVID-19, both because of its uncertain antiviral efficacy and the unclear importance of ongoing viral replication versus an inflammatory process in disease pathogenesis in severely ill patients. Of note, remdesivir yielded positive results in preclinical models of Ebola virus infection but was inferior to two monoclonal antibody therapies in treated patients [15].

During epidemics, as well as calls to treat everyone, other voices demand that vulnerable patients not be treated as ‘guinea pigs’. Clinical trials use well-deliberated, tested, and transparent scientific methods, and are undertaken as a joint endeavour between patients and health professionals within a clear ethical framework. Health emergencies raise additional ethical concerns, but these have been carefully considered by various groups, and clear guidance exists on the conduct of clinical research during health emergencies [20–22].

The choice of the primary endpoint for a clinical trial is always challenging, but it has added difficulties when dealing with a disease with uncertain natural history, an overstretched workforce, and the desire for an early answer. This requires an endpoint that is clinically meaningful, is based on simple data collection, has sufficient power, and will occur early. It also requires statistical methods that are robust to misspecification of design parameters, such as the endpoint frequency. We believe that our primary endpoint is both pragmatic and informative, but we have included a range of secondary endpoints to show consistency with the primary endpoint, as recommended by the US Food and Drug Administration for trials in influenza [23].

### Trial status

The protocol version is number 3.0, dated 9^th^ February 2020.

The first patient, first visit was on 6^th^ February 2020; the recruitment end date was anticipated to be 3^rd^ April 2020.

## Footnotes

1 Acute hepatitis is defined by the following: (1i) Development of ALT ≥ 3 × upper limit of normal (ULN) and bilirubin ≥ 2 × ULN(> 35% conjugated bilirubin). If conjugated/unconjugated bilirubin is not available, urobilinogen should be tested for on a test strip, and a positive result to be indicative of > 35% conjugated bilirubin. If neither tests isare available, the study drug should be discontinued. (2ii) ALT ≥ 5 × ULN. (3iii) ALT ≥ 3 × ULN and onset of symptoms of hepatitis, such as fatigue, nausea, vomiting, right upper quadrant pain, fever, rash, or eosinophilia.
